# Time-scales of hydrological forcing on the geochemistry and bacterial community structure of temperate peat soils

**DOI:** 10.1038/srep14612

**Published:** 2015-10-06

**Authors:** Flavia L. D. Nunes, Luc Aquilina, Jo de Ridder, André-Jean Francez, Achim Quaiser, Jean-Pierre Caudal, Philippe Vandenkoornhuyse, Alexis Dufresne

**Affiliations:** 1 Laboratoire des Sciences de l’Environnement Marin, LEMAR UMR 6539 CNRS/UBO/IRD/Ifremer, Université de Brest (UBO), Université Européenne de Bretagne (UEB), Institut Universitaire Européen de la Mer (IUEM), 29280 Plouzané, France; 2 Université de Rennes 1, CNRS, UMR6118 Géosciences, Rennes, France; 3 Université de Rennes 1, CNRS, UMR6553 ECOBIO, Rennes, France

## Abstract

Peatlands are an important global carbon reservoir. The continued accumulation of carbon in peatlands depends on the persistence of anoxic conditions, in part induced by water saturation, which prevents oxidation of organic matter, and slows down decomposition. Here we investigate how and over what time scales the hydrological regime impacts the geochemistry and the bacterial community structure of temperate peat soils. Peat cores from two sites having contrasting groundwater budgets were subjected to four controlled drought-rewetting cycles. Pore water geochemistry and metagenomic profiling of bacterial communities showed that frequent water table drawdown induced lower concentrations of dissolved carbon, higher concentrations of sulfate and iron and reduced bacterial richness and diversity in the peat soil and water. Short-term drought cycles (3–9 day frequency) resulted in different communities from continuously saturated environments. Furthermore, the site that has more frequently experienced water table drawdown during the last two decades presented the most striking shifts in bacterial community structure, altering biogeochemical functioning of peat soils. Our results suggest that the increase in frequency and duration of drought conditions under changing climatic conditions or water resource use can induce profound changes in bacterial communities, with potentially severe consequences for carbon storage in temperate peatlands.

Water saturation plays a critical role in the maintenance of anoxic conditions and carbon sequestration in soils^1^,^2^, with hydrology influencing the maintenance of soil saturation, anoxic conditions, and ultimately, carbon accumulation in peat. The absence of water saturation such as during droughts can produce significant changes in peatland biogeochemistry. Aerobic conditions following summer drought are often followed by peaks in sulfate (SO_4_^2−^) release, increased acidification and suppression of dissolved organic carbon (DOC) in peatland catchment areas^3^,^4^. Laboratory experiments confirm these field observations^4^,^5^.

Drought conditions stimulate bacterial decomposition of organic matter^6^,^7^. Aerobic conditions also influence the production of CO_2_ and CH_4_, with CO_2_ emissions increasing with water table decline, while CH_4_ production is inhibited^8^. In addition, drought followed by rewetting may alter the availability of electron acceptors, leading to competition for organic substrates and resulting in rate changes or delays in biogeochemical processes such as methanogenesis^9^,^10^. Consequently, hydrological forcing alters the physical and chemical environment, modifying bacterial communities in peat.

Peatlands constitute an important terrestrial carbon reservoir^11^,^12^,^13^, but their role as a carbon sink could be destabilized by environmental change, including changes in hydrology^14^,^15^. Although the influence of hydrology on biogeochemical processes in peat is well documented, in particular with respect to carbon, the long-term effects on microbial communities remain poorly understood. Nevertheless, the extent, nature and time scales of modifications to microbial community structure induced by hydrological forcing are key parameters for assessing anthropogenic impact on regional and global carbon budgets.

The goal of this study is to examine the effects of the hydrologic cycle on the biogeochemistry and bacterial communities in temperate peat at various temporal scales. The peatland in the Cotentin peninsula in Normandy has a well-characterized groundwater budget^16^,^17^, with annual cycles in water table height being superposed by high-frequency daily evapo-transpiration cycles, as well as intermediate-duration variations related to rainfall. In addition, anthropogenic activity, such as well water extraction, not only influences water table height, but also modifies local hydrology, inducing recharge from a surface stream in addition to rainfall^16^.

This study uses a combined geochemical and microbial metagenomic approach within an experimental framework to investigate the following hypotheses: (1) that short-term variations in hydrologic cycle and consequently oxygenation of pore space in peat influences not only pore water geochemistry but also the associated bacterial community and (2) long-term hydrological forcing is able to modify microbial community structure.

Peat cores were taken from two sites separated by 1.5 km, but with distinct hydrological regimes. One site located close to a pumping station has been subjected to more frequent water table drawdown and river water influxes since 1992; a second, pristine site has a more stable water table recharged by rainfall. Peat cores from the pumping station and pristine sites were subjected to four treatments for 63 and 45 days respectively: (1) saturated for 3 days, then unsaturated for 3 days (3-day cycle), (2) saturated for 9 days, then unsaturated for 9-days (9-day cycle), (3) continuously saturated (sat) and (4) continuously unsaturated (dry) (Fig. 1). The 3-day and 9-day cycle periods were chosen to represent short-term fluctuations in water table height, typically related to evapo-transpiration and episodes of rainfall, whilst the pumping station and pristine site comparison allows testing the impact of hydrologic variations on a decadal time scale. Water was sampled from the cores at three time points over the course of the experiment in order to examine the temporal changes in both geochemical parameters and microbial community structure. Microbial community was also assessed for soil samples from (1) the two field sites (pumping station and pristine), and (2) from the experimental cores at the end of each experiment. Microbial community composition and diversity was assessed via metagenomic profiling of the microbial 16S ribosomal RNA gene. A second set of experiments, where peats from each site were subjected to similar saturation/desaturation cycles after being desiccated for 5 months without any rewetting, examined changes in biomass and geochemistry. Multivariate statistics were used to describe patterns in microbial community structure, and to make associations among biological results and geochemical data.

## Results

### Effect of hydrology on biogeochemical processes

Multivariate geochemical analysis of the pore waters collected during the dry-rewetting experiments identified both acidification and redox processes in the pumping station site. These include pH decrease correlated to SO_4_^2−^ increase and cation leaching (acidification), reduced availability of DIC and DOC, lower ion concentrations and less intense denitrification (redox processes) (Geochemical Analysis, Supporting Information). More reducing conditions, including high Fe and very low NO_3_, were observed in the permanently saturated cores. In contrast, cores subjected to 3-day and 9-day dry-rewetting cycles were characterized by less reducing conditions. Indeed, the intensity of denitrification of the saturating groundwater decreases with cycle duration, with denitrification being most efficient in the permanently saturated cores and least efficient in the 3-day cycle cores (Fig. 2, Geochemical Analysis, SI).

Peat cores from the pumping station site presented more intense acidification processes, had lower DOC and DIC and higher leaching of ionic species, while DOC and DIC remained high in peat cores from the pristine site, irrespective of dry-rewetting cycle periodicity (Fig. 2A). The cores from the pumping station site also showed lower total biomass than from the pristine site with the notable exception of dry cores in the most oxic conditions (Geochemical Analysis, SI).

### Effect of hydrology on microbial diversity

Taxonomic profiles of water collected from the cores and soil samples collected during the experiment are shown in Fig. 3A. Among the 14 most common phyla, significant differences (*p* < 0.01) are found in the average abundances of 8 phyla between the water and soil samples. Water samples are characterized by higher percentages of sequences assigned to β-Proteobacteria, γ− Proteobacteria, Bacteroidetes and Actinobacteria (2.1x, 4.5x, 2.6x and 2.7x greater in water samples respectively), while soil samples were richer in sequences assigned to Acidobacteria, Chloroflexi, Nitrospirae, Chlorobi and Spirochaetes (3.3x, 2.1x, 1.9x, 2.0x and 23.5x greater in soil samples respectively). Phyla abundances were also significantly different (*p* < 0.01) in soil samples from the pristine and pumping station sites, where Acidobacteria and Actinobacteria were greater in the pumping station site (1.6x and 2.1x greater respectively), while α-, γ- and δ-Proteobacteria as well as Chloroflexi and Planctomycetes were more abundant in the pristine site (2.2x, 2.6x, 2.4x, 1.8 and 3.7x greater, respectively).

Bacterial community diversity is shown in Fig. 3B and Fig. S1. General linear models (GLM) were used to determine which factors (among sampling site, cycle period, and sampling time) had a significant impact on diversity. For soils, sampling site (pumping station vs. pristine) was a significant factor for all diversity indices (*p* < 0.001), with diversity being lower in the pumping station site. For water collected from the cores, richness was significantly lower in the pumping station site (*p* < 0.001), as well as in the groundwater samples (*p* < 0.001) (Fig. 3B), but differences among sites were less pronounced in Shannon diversity and Equitability (Fig. S1). Dry-wet cycle periodicity (3-day, 9-day and saturated) significantly impacted Shannon diversity (*p* < 0.001) and Equitability (*p* < 0.001) in water collected from the cores.

Non-metric multidimensional scaling (NMDS) based on Bray-Curtis similarity revealed that the bacterial communities in core-collected water, soils and groundwater are clearly distinguished among one another and between the pristine and pumping station sites (Fig. 4). Principal component analysis of the geochemical data indicates that the pristine site is characterized by higher concentrations of DOC and DIC, while the pumping station site has higher concentrations of dissolved ions (Fig. 2) and lower total and active biomass (Geochemical Analysis, SI). The concentration of key geochemical parameters overlain on the NMDS plots of bacterial community from the pristine and pumping station sites indicates a correlation among geochemistry and community structure (Fig. 5). Pearson rank correlation among the best combination of geochemical variables and Bray-Curtis pairwise resemblance (BIONENV test in Primer 6) finds that SO_4_^2−^, DOC and Si best describe differences between pristine and pumping station sites (*p* = 0.002).

Within each site, community structure in the water samples evolved over the course of the experiment, such that the first two sampling time points are distinguished from the final sampling time point, and sampling times have greater similarity than treatments (not shown). To exclude the effect of time, dry-wet cycle periodicity was only considered for the final time point. Bacterial community structure in the core waters was found to be similar among the 3-day and 9-day cycles for both sites (Fig. 6A,B) with overlapping pore water geochemistry characteristics (Fig. 2B,C). In contrast, the continuously saturated cores had divergent bacterial communities (Fig. 6A,B), with the pore water geochemistry being characterized by greater DOC, Fe and Al in the saturated cores in both sites, with the exception of one divergent core in the pumping station site (Fig. 2B,C). The BIOENV test, which tests for correlations between environmental variables and Bray-Curtis similarity of bacterial communities, did not show any significant correlation in the pristine site (*p* = 0.17), but for the pumping station site a significant correlation was observed between environmental and bacterial community data (*p* = 0.016), with Na, Al, Ca, Si and Fe most distinguishing the 3-day + 9-day cores from the saturated cores. In the “dry” cores, total and active biomass was much lower than the 3-day, 9-day and saturated cores in the pristine site, while the opposite was observed in the cores from the pumping station site (Geochemical Analysis, SI).

For soils, although bacterial communities were distinguished by site, with the soil communities in the pumping station site being clearly distinguished from the pristine site (Fig. 6C), they were not distinguished by cycle periodicity (3-day, 9-day, sat, dry) or time (Beginning and End of Experiment) (Fig. 6D), suggesting that neither cycle periodicity or sampling time had an effect on soil microbial community structure.

## Discussion

Striking differences in the geochemistry and bacterial communities are observed between peat soils and waters from a pristine site and a site adjacent to a water pumping station in a peatland in Normandy. The two sites are separated by only 1.5 km, and despite heterogeneity in peat^18^, it is unlikely that plant and mineral types within the same depositional basin would differ greatly at this scale. Replication of experimental cores also ensures that localized heterogeneity at the level of meters has been averaged, and that the geochemistry and community structure measured are representative of each site and treatment. On the other hand, both sites are known to have different hydrology, the pumping station site having experienced greater variability in volume and source of recharge, and the pristine site having more stable groundwater fluxes^16^,^17^. We therefore propose that the observed differences in geochemistry and bacterial community composition are primarily driven by hydrologic forcing. The peat core experiments conducted under controlled conditions, on the other hand, provide insights on the effect of hydrology at shorter time-scales, of the order of several days to weeks.

### Community structure in groundwater, core-collected water and soils, and exchange between peat matrix and pore water

Bacterial communities in the groundwater used to saturate the experimental cores, water collected by draining each core, and soils extracted from the cores were highly distinguished from one another (Figs 3,4,6, S1). The groundwater used to saturate the pumping station cores was found to have different community composition than that used for the pristine cores (Fig. 4), although the water was sampled at the pumping station for both experiments. This finding is likely the result of extracting water at different states of the hydrological cycle for the two experiments. Temporal variation in groundwater bacterial communities has been observed in this study site^19^ and elsewhere^20^. Interestingly, water communities were more diverse (Fig. 3) and more similar to soil communities than to the groundwater used to saturate the cores (Fig. 4). Groundwater is characterized by low diversity, and is likely to have low bacterial abundance; therefore, its addition to the peat likely has little effect on bacterial community structure of the peat waters. Rather, the composition of the groundwater is modified shortly after coming into contact with the peat matrix, possibly representing a mix of the groundwater and peat soil community. The NMDS analysis supports this interpretation, as the peat waters have an intermediate degree of similarity between the groundwater and soil communities (Fig. 4).

Microbial communities in water collected from the cores appear to be more dynamic than soil, with community structure evolving over time and in response to experimental treatments (Fig. 6), possibly in response to availability of new substrates following changes in the chemical environment, or by release from the soil compartment. Conversely, soil bacterial communities appear to be fairly stable despite being subjected to repeated groundwater saturation cycles. Indeed, soil communities did not differ depending on the cycle treatment, nor did they change over the course of the experiment (Fig. 6D). These observations are likely related to high bacterial abundances in soils which moderate change, as well the attached lifestyle of many microbes onto soil particles^21^. In addition, the forces that allow bacterial attachment to soil particles to take place^22^ may contribute to the stability of soil communities.

The observed patterns in bacterial community structure in the three compartments, groundwater, core water and soils, and geochemical analysis (SI) are consistent with a dual-porosity system described for solute transport, where peat is composed of interconnected pores that actively transmit water and dead-end and closed pores included in the peat matrix which retard flow^23^ (Fig. 7). The interconnected pores are open to external fluxes of water and are associated with dynamic changes, such as the groundwater communities becoming more similar to soil communities once they enter the peat soil. The poorly connected pores and peat matrix are characterized by diffusive flow, leading to slower processes that are intimately associated to the peat matrix, in more reducing conditions. The closed pores that constitute the preferential habitat for microbes are therefore the core of the biogeochemical reactor (Fig. 7).

Repeated oxygenation of peat soils leads to a progressive loss of peat functionality, such as denitrification, as a consequence of less reducing and more acidic conditions. Oxygenation induces a loss of biomass and diversity. Microbial communities become less diverse and microorganisms adapted to more acidic conditions (Acidobacteria) become more abundant. In soil environments, spatial and temporal heterogeneity are often invoked to explain high levels of diversity in bacterial communities^21^,^24^. Peat soils are highly heterogeneous, and have expected high levels of bacterial richness and diversity^25^. In boreal peats, water-level drawdown has been shown to have a significant impact on microbial community structure^26^, although the effects of drainage and desiccation vary depending on the type of peat and microbial group studied. For example, the diversity of Actinobacteria can be negatively or positively impacted by water-level drawdown, depending on the type of peat studied^26^, and the effects of water-level drawdown on community structure were more apparent in a nutrient-rich fen than a nutrient-poor bog^27^. Vegetation type and litter quality are important additional factors influencing microbial diversity in peats, including in bacteria and fungi^25^,^27^.

Soil pH has been correlated with bacterial diversity in a wide range of soils^28^,^29^,^30^, with acidic soils typically having lower bacterial diversity^28^. Although pH was not directly measured in the pore waters in our experiments, lower pH has been found in the pumping station site relative to the pristine site^17^,^31^, and the sulfate and cation leaching observed in the pumping station site is characteristic of acidified soils. Reduced bacterial diversity in the pumping station site is therefore in agreement with patterns of diversity observed in other acidified soils. Although higher bacterial diversity has been associated with drier conditions in some soils^32^, our experiments show reduced bacterial richness and diversity in samples subjected to drier conditions (Fig. 3, S1). In peat, recurrent drought and rewetting may lead to cracking and widening of preferential flow paths in the porous medium, making resources more accessible to competitive bacteria^21^,^24^, which can then become dominant, thereby reducing diversity. Indeed, most microbial groups are sensitive and not resilient to environmental change^33^, with strong and frequent disturbance being associated with bacterial diversity loss in soils^34^. Furthermore, previous field observations in boreal peats^26^,^27^,^35^ indicate that there can be variability of microbial communities in response to changes in water-level drawdown depending on the peat type. The generality of the impacts of drought-rewetting on the microbial diversity and community structure of various types of northern latitude peats needs to be further explored. Nevertheless, our results point towards important changes in biogeochemistry and bacterial community structure as a result of hydrological forcing, at least in some types of peat.

### Time-scales of the effects of hydrology on community structure

Our experiments show a significant difference in the geochemistry and bacterial community structure of peats submitted to dry-rewetting cycles compared to continuously saturated peats. However, bacterial communities subjected to different short-term cycles (3- or 9-day cycles), do not differ significantly (Fig. 6). This suggests that oxygenation of short duration can alter the redox environment, but that drought duration greater than the tested 9 days is required to significantly modify bacterial communities. In the dual porosity reservoir concept (Fig. 7), peat soils can support rapid and frequent drying and/or influx of oxygen-rich water because many pore spaces are isolated and only subjected to slow diffusive processes, allowing maintenance of microbial community structure and only limited changes in biogeochemical function. The comparison between the pumping station site and pristine sites shows that frequency and duration of drought conditions matter, with repeated and longer time periods of water table drawdown (months) having a greater effect on geochemistry and bacterial communities of peat soils than shorter time scales (days to weeks). This study shows that areas subject to frequent oxygenation and desiccation are more likely to experience acidification processes including degradation of organic matter followed by changes in pore water geochemistry such as leaching of sulfate, iron and other ionic species, as well as changes in ecosystem functioning, as a result of shifts in bacterial community structure. Previous work, which examined groundwater extracted directly from the peat aquifer from the same pumping station and pristine sites, found that peat groundwater from the pumping site is characterized by higher nitrate and sulfate concentrations and lower pH^17^. Furthermore, a follow-up experiment where the same peat cores were exposed to a 5-month desiccation period (SI and^31^) showed sharp acidification effects, especially in the cores from the pumping site. Altogether, these data suggest that long droughts constitute a progressive phenomenon that might irreversibly modify the geochemistry and bacterial communities of peat within decadal time scales.

In light of changing global climate patterns, increase in frequency and duration of drought conditions may have serious consequences for carbon storage in peatlands. Likewise, increased pressure on water resources may mean that extraction from wetland aquifers could lead to longer periods of soil desiccation. Wetlands are currently a carbon sink, but changing environment and land use practices may change this, highlighting the need for preservation of this important ecosystem.

## Methods

**Figure 1:**
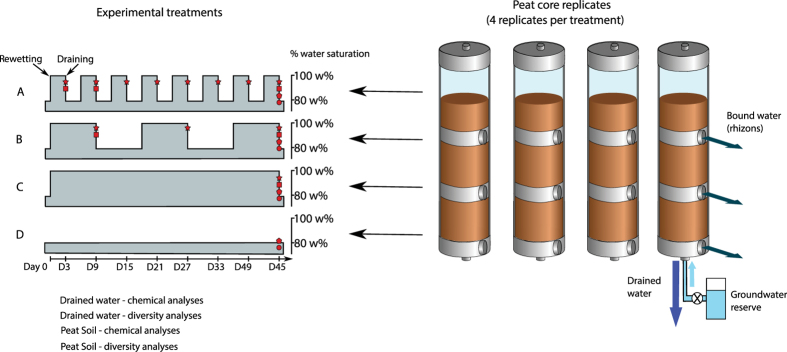
Sketch of the experimental set-up for the dry-rewetting experiment. (**A**) 3-day cycle; (**B**) 9-day cycle; (**C**) continuously saturated; (**D**) continuously unsaturated.

**Figure 2:**
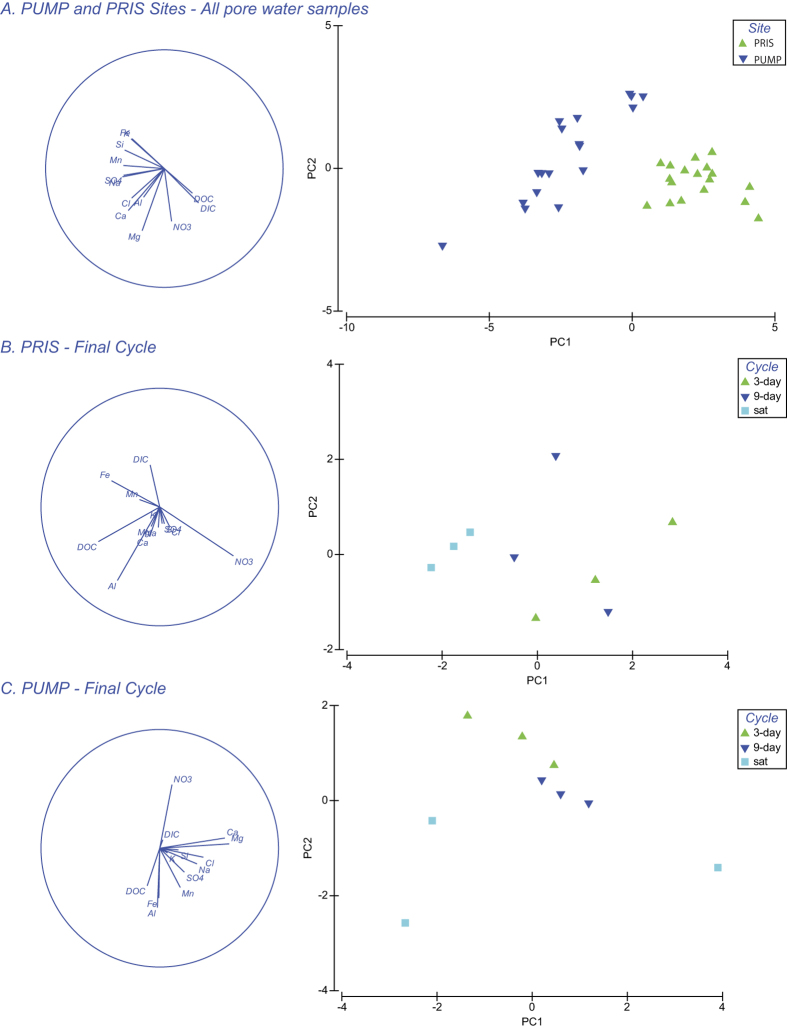
Principal Component Analysis of pore water geochemistry, showing eigenvector plots (left column) and PCA scatter plot (right column) for comparison between (A). All pore water samples collected in the pumping station site (PUMP) and pristine site (PRIS) over the course of the experiment; (**B**) Pore water samples collected in the pristine site (PRIS) at the end of the experiment (Final Cycle); (**C**) Pore water samples collected in the pumping station site (PUMP) at the end of the experiment (Final Cycle).

**Figure 3:**
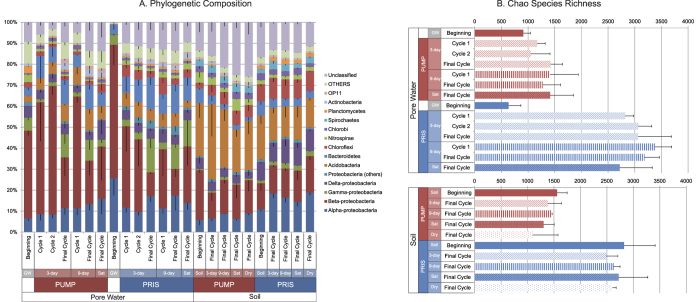
Bacterial community composition and diversity in water and soil samples collected from the pumping station site (PUMP), pristine site (PRIS) and groundwater (GW) used to saturate the experimental cores. (**A**) Phylogenetic composition, shown as the percentage of the ten most abundant phyla, with the remaining 27 phyla grouped under “Others”. Proteobacteria have been further subdivided into α-, β-, γ-, δ-Proteobacteria and others (ε-, TA18 and unclassified). Unclassified OTUs had no match with the Silva Bacterial Database. (**B**) Chao species richness. In both panels, each bar represents the average of 3 replicate samples with the standard error shown as line inside or above each bar.

**Figure 4:**
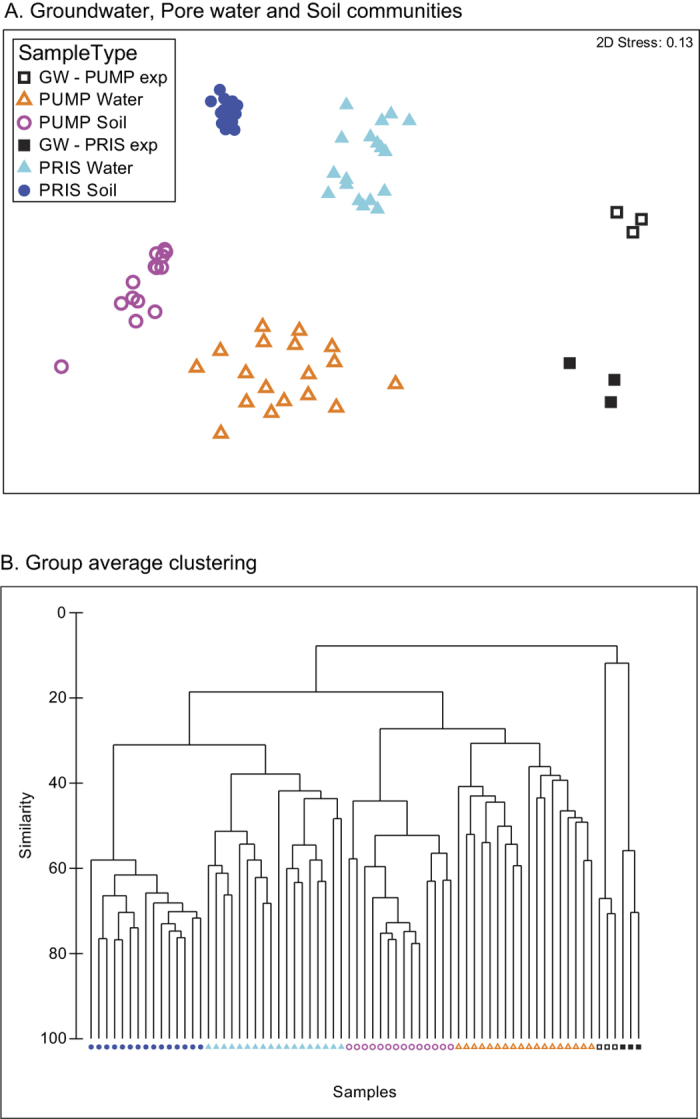
(**A**) NMDS of bacterial communities based on Bray-Curtis similarities. Bacteria collected from the pristine (PRIS) and pumping station (PUMP) sites were sequenced from water extracted from cores (Water), from soil collected from the field site and from cores at the end of the experiment (Soil), and from groundwater (GW) used to saturate cores during the PRIS and PUMP experiments. Groundwater, pore water and soil community are clearly distinguished from one another, as well as by sampling site. (**B**) Group-average clustering dendrogram based on Bray-Curtis similarities.

**Figure 5:**
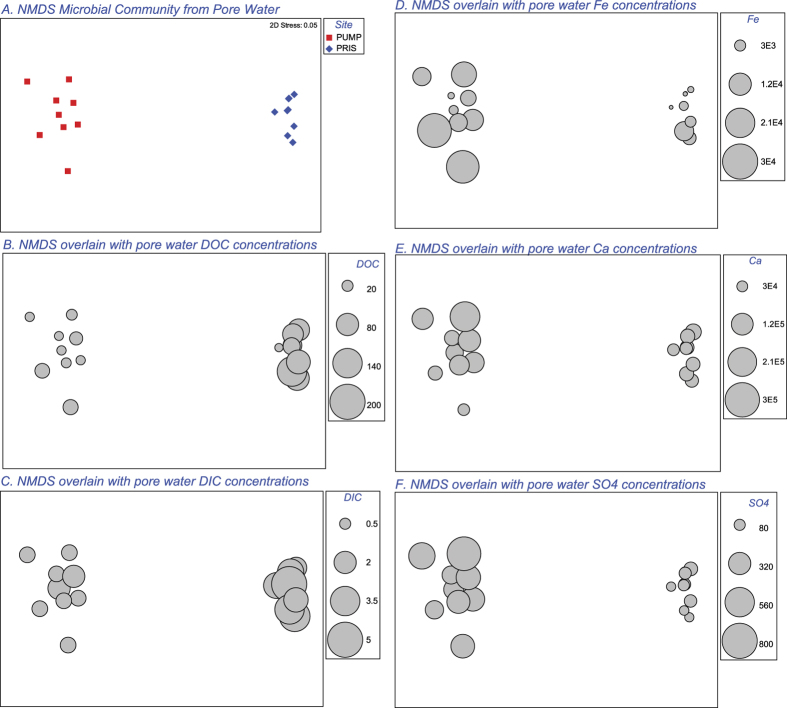
(**A**) NMDS based on Bray-Curtis similarity of bacterial communities in core-collected waters of the pristine (PRIS) and pumping station (PUMP) sites, collected at the end of the experiment. Panels (**B**–**F**) show the NMDS overlain with circles proportional to the concentration of dissolved organic carbon (DOC), dissolved inorganic carbon (DIC), total iron (Fe), calcium (Ca^2+^) and sulfate (SO_4_^2−^) measured in the pore waters at the time of sampling. The PRIS site has high levels of DOC and DIC relative to the PUMP site, while the PUMP site is characterized by high levels of Fe, Ca^2+^ and SO_4_^2−^.

**Figure 6:**
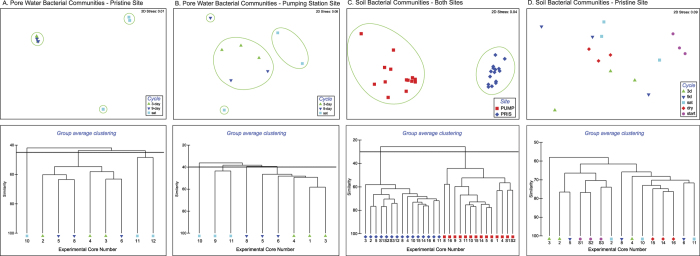
NMDS of bacterial communities in pore water and soil based on Bray-Curtis similarity. Green ellipses indicate group average clustering of samples for the similarity threshold shown in the dendrogram below. Panels (**A**,**B**) show samples subjected to three experimental dry-rewetting cycles (3-day, 9-day and saturated) taken at the end of the experiment (Final Cycle). Most communities subjected to 3-day and 9-day dry-rewetting cycles fall within a cluster, while bacterial communities in continuously saturated cores are distinct from the 3-day and 9-day cycles, and at times distinct from other saturated cores, indicating high variance among communities in saturated cores. Panels (**C**,**D**) show soil bacterial communities. Soil communities in the pristine site (PRIS) are distinct from communities in the pumping station site (PUMP) (Panel (**C**)), while within the prisitne site, there is no clear evidence of an effect of dry-rewetting cycle (Panel (**D**)). A similar pattern is observed in the pumping station site (data not shown).

Peat samples were taken from two sites in the Parc Naturel Regional des Marais du Cotentin et du Bessin, Normandy. The pumping station site was located in the vicinity of a groundwater well in La Bergerie (49°13'25.72″N, 1°21'50.70″W). A pristine site was chosen approximately 1.5 km downstream (49°14'11.97″N, 1°21'24.19″W) in an undisturbed area. Sixteen sediment cores were collected at each site at 50 cm below the sediment surface. The peat cores were subjected to four experimental treatments, each with four replicate cores, where peat core water saturation varied by (1) 3 days saturated – 3 days unsaturated, (2) 9 days saturated – 9 days unsaturated, (3) continuously saturated and (4) continuously unsaturated (Fig. 1). Peat cores from both sites were saturated using groundwater from the extraction well in the pumping station site, characterized by higher nitrate concentrations (40 mg/L) and oxidizing characteristics than peat pore water. Each saturation cycle thus represents a perturbation of the reducing conditions characteristic of the peat medium. Groundwater was chosen to saturate the peats in order to reproduce conditions observed in the field. Both sites are part of the same drainage basin and are underlain by the same aquifer. Previous work that quantified water fluxes in the study area shows that groundwater partly saturates the peat at both sites each year during the high water period^16^,^17^ and a geochemical investigation of the whole aquifer showed relatively homogeneous groundwater composition^31^. Groundwater was collected from the pumping station before each dry-rewetting experiment, such that the water used was fresh and had geochemical composition and microbial community representative of the time of sampling.

A second set of experiments (“desiccation experiment”) was carried out using peat cores from the same sites and similar experimental conditions, only peat cores were kept at the lab temperature without any water addition during 5 months before the experiment. After the 5 months, the water content was 60 w% instead of the initial 80 w%. Only geochemical analyses and biomass quantification were carried out for this experiment.

**Figure 7:**
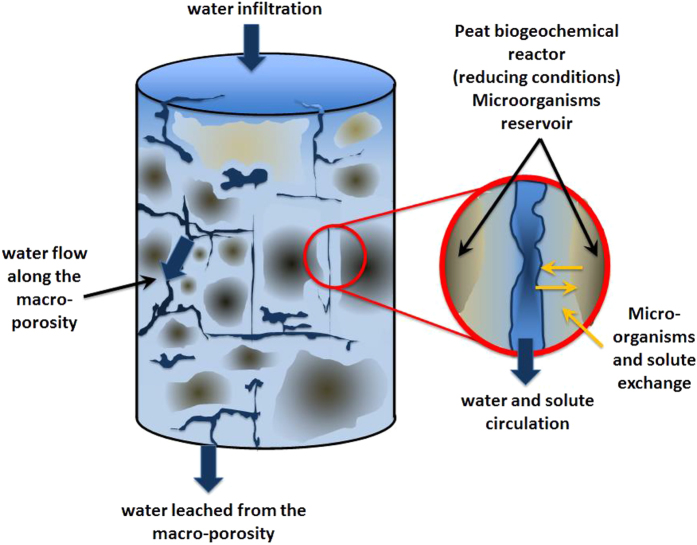
Sketch of the dual-porosity concept of the peat soil and associated biogeochemical reactor. The peat soil is composed of interconnected pores that actively transmit water and dead-end and closed pores associated to the peat matrix which retard flow. The closed pores constitute the preferential habitat for microbes are therefore the core of the biogeochemical reactor.

At the end of each cycle period, water drained from the cores was collected for geochemical analysis, as was pore water from the middle of the core, using 0.10 μm Rhizon samplers. The water drained from the cores represents water from the interconnected pores, while water collected with the Rhizon samplers represents water from the closed and dead-end pores (Fig. 7). In the text, the term pore water refers to water sampled with the Rhizon samplers, while core-collected waters refers to water drained from the cores at the end of each saturation cycle. Dissolved Organic Carbon (DOC) and Dissolved Inorganic Carbon (DIC) were measured with a Total Organic Carbon analyzer (Shimadzu TOC-5050A). Major anions concentrations (Cl^−^, NO^3−^, NO^2−^, PO_4_^2−^, SO_4_^2−^) were measured by ion chromatography (Dionex DX-120), and major cation concentrations (Na^+^, Mg^2+^, K^+^, Ca^2+^, Mn^2+^ and Fe_TOTAL_) were measure by ICP-MS (Agilent 4500), using indium as an internal standard. The international geostandard SLRS-4 was used to check the reproducibility of the results. Fe (II) concentration was measured by the 1.10 phenantroline colorimetric method using a UV visible spectrophotometer (AFNOR, 1997) and Fe (III) was calculated by subtracting Fe(II) from Fe_TOTAL_. Total and active microbial biomass were measured on 4 peat samples before and at the end of the experiment. For total biomass, a modified fumigation-extraction method specific for peat was used^36^. Active biomass was measured with the substrate-induced respiration method, by adding 18 mg/g of glucose and measuring the respiration rate (CO_2_ release) after 1 hour incubation by gas chromatography^37^.

For molecular analysis of bacterial communities, water was drained from the cores with a peristaltic pump from 3 of the 4 replicate cores after the first 3-day cycle, after the first 9-day cycle, and at the end of the experiment. Three replicate samples of the groundwater used to saturate the experimental cores were collected at the beginning of the experiment. A volume of 60 mL was filtered through 0.22 μm Sterivex filters (Millipore). Filters were flash frozen in liquid nitrogen and kept at −80 °C. Peat samples were collected from the sampling site, and from the center of each core at the end of the experiment. Environmental DNA was extracted using the Power Soil DNA Extraction kit (MoBio Laboratories), with a bead-beating step of 2 minutes at 30 Hz, using the Retsch MM400 mixer mill.

Amplicon libraries were prepared using fusion primers that contained the typical Adaptor A or B (Lib-A protocol), a multiplex identifier (5 or 10 bases in length) and the specific primer sequence. The forward (5′-GTG CCA GCM GCC GCG GTA ATA C-3′) and reverse (5′-CCG TCA ATT CCT TTG AGT TT-3′) primers amplify a fragment spanning the V4 and V5 regions of the ribosomal 16S gene. Amplification using the AmpliTaq PCR kit (Sigma) had an initial denaturation at 94 °C for 2 min; 30 cycles of denaturation at 94 °C for 30 s, annealing at 64 °C for 30 s and extension at 72 °C for 60 s, and a final extension for 6 min. Amplicon libraries were purified using the Agencourt AMPure XP magnetic beads and quantified by fluorometry with the Quant-iT PicoGreen dsDNA kit (Invitrogen). Amplicons were multiplexed and sequenced with a Roche GS-FLX Titanium pyrosequencer at the Environmental Genomics platform of the Observatoire des Sciences de l’Univers de Rennes (University of Rennes I, France). Every sample was sequenced as two biological replicates, starting from independent amplicons.

Quality screening of the pyrosequencing results removed reads that: (1) had a length shorter than 400 bp, (2) had any ambiguous base pair assignment or (3) singletons present in only 1 sample. Singletons present in at least 2 samples were kept in the analysis, but identical reads present in only one sample were excluded. Chimeric sequences were identified by uchime in Mothur^38^ and removed from the dataset. Following quality screening, 1,527,190 reads were recovered (SuppTable1).

DNAclust^39^ was used for defining operational taxonomic units (OTUs) at 97% sequence identity, which were then classified by comparison with the Silva ribosomal RNA databases for Bacteria and Archaea^40^ in Mothur^38^. Taxonomic profiles consisting of the percent composition of major bacterial phyla were generated for each sample. Rarefaction curves were calculated for each sample in Mothur^38^.

A matrix containing the total count of each OTU in each sample was generated using an in-house Perl script. OTU counts were standardized to a frequency. The following diversity indices were calculated for each sample: (1) Chao 1 species richness; (2) Shannon diversity index and (3) Shannon Equitability index. General linear models implemented in R were used to identify factors that were statistically significant among sites, treatments and sampling times. Significant differences in average abundance of phyla was calculated with Student’s t-test in R. A log(x+1) transformation was applied to all OTU relative frequencies. Ordination analyses were performed by non-metric multidimensional scaling (NMDS) using the Bray-Curtis similarity coefficient in Primer6^41^. NMDS generates a scatter plot in which the configuration of points relates the ranks of a similarity matrix as Euclidean distances. In short, on an NMDS plot, similar samples are plotted closely together, while dissimilar samples have greater distances between them.

Geochemical measurements for pore water data were log(x+1) transformed and normalized. Principal Component Analysis was used to reduce the dimensional complexity of the dataset and examine the contributions of each variable to the variance of the dataset. Associations between environmental and biological data were examined in two ways. Pore water concentrations of the most distinguishing variables identified by PCA were overlain on the NMDS plots. This allowed similarity in environment and in bacterial community structure to be examined. In addition, the BIOENV procedure implemented by Primer 6 was used to determine which combination of environmental variables resulted in the best rank correlation coefficients with respect to the sample similarity matrix for the microbial communities^41^. 500 permutations were performed to test statistical significance of correlations between biotic and abiotic matrices.

## Additional Information

**How to cite this article**: Nunes, F. L. D. *et al.* Time-scales of hydrological forcing on the geochemistry and bacterial community structure of temperate peat soils. *Sci. Rep.* **5**, 14612; doi: 10.1038/srep14612 (2015).

## Supplementary Material

Supplementary Information
