# Supplementary material for: Time-scales of hydrological forcing on the geochemistry and bacterial community structure of temperate peat soils
Source: Sci Rep. 2015 Oct 6;5:14612. doi: 10.1038/srep14612 (PMC4594127; doi:10.1038/srep14612)
Supplement: Supplementary Information [file srep14612-s1.pdf]

Time-scales of hydrological forcing on the geochemistry and bacterial community structure of temperate peat soils

Flavia LD Nunes, Luc Aquilina, Jo de Ridder, Andre-Jean Francez, Achim Quaiser, Jean-Pierre Caudal, Philippe Vandenkoornhuyse and Alexis Dufresne

SUPPORTING INFORMATION

Geochemical analysis

PCA analysis of the pore water from the core experiments showed that the geochemical composition is mainly controlled by two major process types[26]. The first axis mainly characterizes acidification processes with low pH on one side opposed to high sulfate and cation concentrations. These high concentrations are related to reduced sulfur and/or organic matter dissolution. High cation concentrations are related to acidification neutralization through mineral dissolution. PCA shows a clear distinction between the pumping and the pristine site, whatever the cycle conditions, the pumping site showing more acid and less reducing conditions. Within one site, the cycle duration also induces a shift in the geochemical conditions with more acid conditions in the 3d cycle. Indeed low to extremely low (3.2) pH and high to extremely high (>1,000mg/L) sulfate concentrations were observed in the field and within the core experiments. They were particularly well expressed in the "dessication experiment" in the lab and during rewetting processes in the field[18]. The second axis is related to oxydo-reduction processes, showing on one side oxygenated conditions with high nitrate concentrations opposed to high Fe and DOC concentrations. This part of the axis represents the reducing conditions of the peat, especially in the inner part of the cores (the peat matrix) whilst the opposite part of the axis represents the part of the peat core which are in contact to groundwater and atmosphere such as the upper part of the cores and the leaching water.

The total biomass in the pristine site was much higher than in pumping station experiments (750 and 200 µgC/g dry soil respectively in the permanently saturated and cycle column). Furthermore, the biomass was higher in the dry cores (600 µgC/g dry soil) than in the drying / rewetting cycle cores in the pumping station site. Within the pristine site, the biomass remains relatively similar in the cycling conditions and the permanently saturated cores whilst it is lower in the dry cores (400 µgC/g dry soil). This result suggests that the microbial communities could be better adapted to reducing conditions in the pristine site and better adapted to aerobic and acid conditions in the pumping station, suggesting a microbial shift between the two sites.

The experiments carried on the peat cores after the desiccation period showed a major chemical evolution as compared to the previous ones[26]. A dramatic acidification was characterized by a pH shift (down to 3.8), very high sulfate concentrations (>1,700 mg/L) and a cation increase. The total biomass was also 3 times more important than during the previous experiments, suggesting a major shift in the biodiversity.

Figure S1. Shannon Diverisity and Equitability indices for bacterial communities in pore water and soil from the pumping station site (PUMP) and pristine site (PRIS). Each bar shows the average index and standard error calculated for 3 replicate cores. The groundwater (GW) used to saturate the experimental cores was sampled at the beginning of the experiment for each site. Pore water drained from the cores was collected after the first two 3-day cycles (Cycles 1 and 2 for the 3-day treatment), after the first 9-day cycle (Cycle 1 for 9-day treatment), and at the end of the experiment for the 3-day, 9-day and continuously saturated treatments (Final Cycle). For soil, a small soil sample was collected in the field in the same location where cores were collected (Beginning). At the end of the experiment (Final Cycle), cores were cut open and soil samples were taken from the middle of the core for the 3-day, 9-day, continuously saturated (Sat) and continuously unsaturated (Dry) treatments.

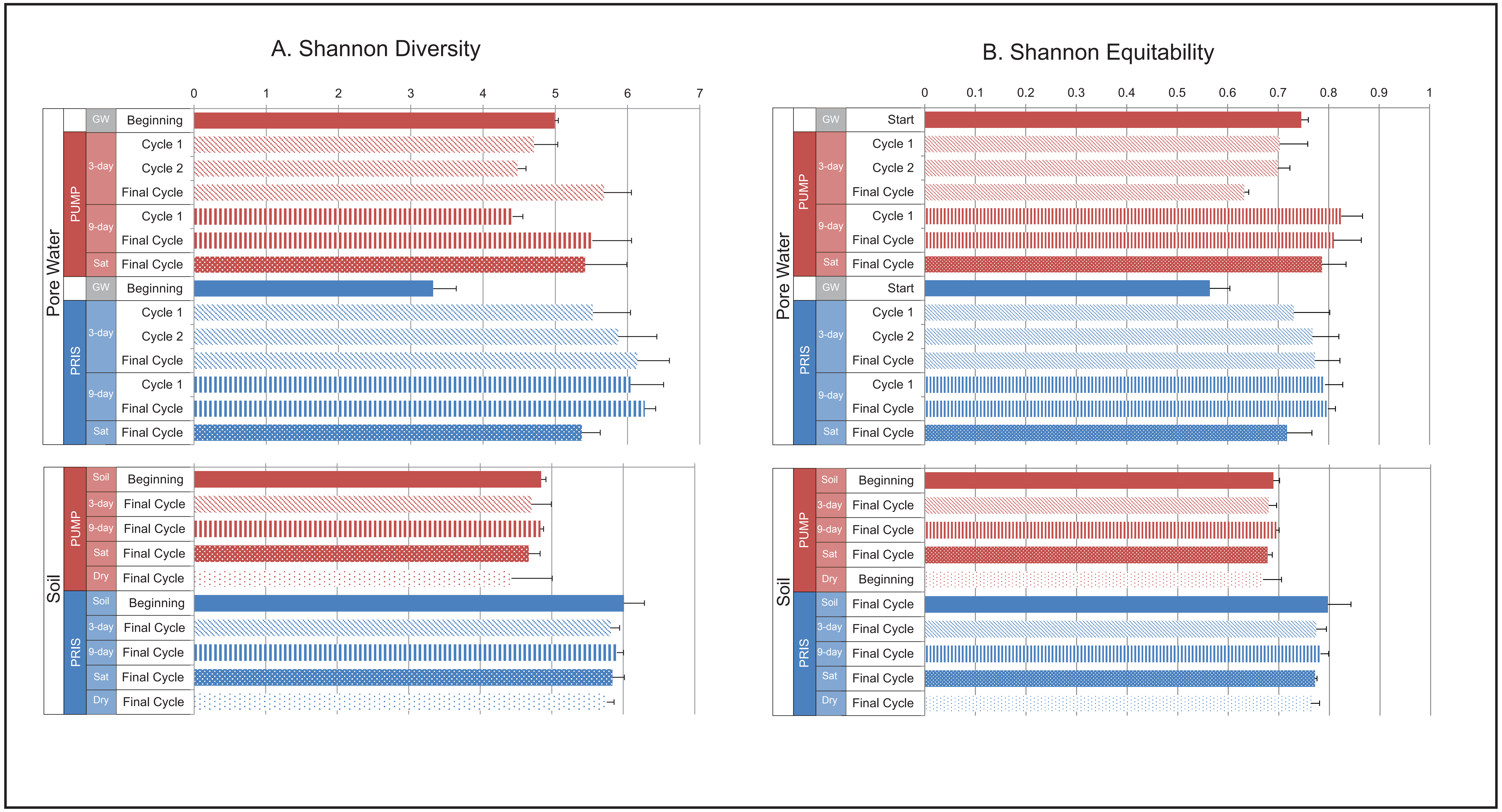

Supplementary Table 1. Sample information and sequencing statistics, including number of reads and OTUs per replicate

Sample ID	Pore Water or Soil	Site	Dry-Wet Cycle	Sampling Day	Time	Core Number	Number of Reads	Number of OTUs
Ex1Eau1	Pore Water	PUMP	groundwater	1	Beginning		37874	714
Ex1Eau2	Pore Water	PUMP	groundwater	1	Beginning		35100	946
Ex1Eau3	Pore Water	PUMP	groundwater	1	Beginning		25522	790
Ex1T11	Pore Water	PUMP	3-day	4	Cycle1	1	11855	733
Ex1T12	Pore Water	PUMP	3-day	4	Cycle1	3	6372	760
Ex1T13	Pore Water	PUMP	3-day	4	Cycle1	4	22054	960
Ex1T21	Pore Water	PUMP	3-day	10	Cycle 2	1	6569	592
Ex1T23	Pore Water	PUMP	3-day	10	Cycle 2	3	8308	777
Ex1T24	Pore Water	PUMP	3-day	10	Cycle 2	4	4499	480
Ex1T25	Pore Water	PUMP	9-day	10	Cycle1	5	28188	987
Ex1T26	Pore Water	PUMP	9-day	10	Cycle1	6	35265	1515
Ex1T28	Pore Water	PUMP	9-day	10	Cycle1	8	26115	801
Ex1Tf1	Pore Water	PUMP	3-day	63	Final Cycle	1	5914	974
Ex1Tf3	Pore Water	PUMP	3-day	63	Final Cycle	3	5659	1104
Ex1Tf4	Pore Water	PUMP	3-day	63	Final Cycle	4	5832	844
Ex1Tf5	Pore Water	PUMP	9-day	63	Final Cycle	5	10128	1146
Ex1Tf6	Pore Water	PUMP	9-day	63	Final Cycle	6	5312	931
Ex1Tf8	Pore Water	PUMP	9-day	63	Final Cycle	8	8061	676
Ex1Tf9	Pore Water	PUMP	saturated	63	Final Cycle	9	8970	921
Ex1Tf10	Pore Water	PUMP	saturated	63	Final Cycle	10	6183	715
Ex1Tf11	Pore Water	PUMP	saturated	63	Final Cycle	11	9705	1337
Ex2Eau1	Pore Water	PRIS	groundwater	1	Start		17884	258
Ex2Eau2	Pore Water	PRIS	groundwater	1	Start		20304	319
Ex2Eau3	Pore Water	PRIS	groundwater	1	Start		13643	531
Ex2T12	Pore Water	PRIS	3-day	4	Cycle 1	2	15836	1843
Ex2T13	Pore Water	PRIS	3-day	4	Cycle 1	3	17903	1967
Ex2T14	Pore Water	PRIS	3-day	4	Cycle 1	4	16059	1929
Ex2T22	Pore Water	PRIS	3-day	10	Cycle 2	2	19214	2025
Ex2T23	Pore Water	PRIS	3-day	10	Cycle 2	3	14142	1775
Ex2T24	Pore Water	PRIS	3-day	10	Cycle 2	4	21425	2495
Ex2T25	Pore Water	PRIS	9-day	10	Cycle 1	5	23194	2152
Ex2T26	Pore Water	PRIS	9-day	10	Cycle 1	6	22821	2648
Ex2T28	Pore Water	PRIS	9-day	10	Cycle 1	8	25074	2627
Ex2Tf2	Pore Water	PRIS	3-day	45	Final Cycle	2	23862	2684
Ex2Tf3	Pore Water	PRIS	3-day	45	Final Cycle	3	26464	2504
Ex2Tf4	Pore Water	PRIS	3-day	45	Final Cycle	4	18301	1756
Ex2Tf5	Pore Water	PRIS	9-day	45	Final Cycle	5	23906	2238
Ex2Tf6	Pore Water	PRIS	9-day	45	Final Cycle	6	23630	2567
Ex2Tf8	Pore Water	PRIS	9-day	45	Final Cycle	8	29343	2593
Ex2Tf10	Pore Water	PRIS	saturated	45	Final Cycle	10	27652	2442
Ex2Tf11	Pore Water	PRIS	saturated	45	Final Cycle	11	12384	1748
Ex2Tf12	Pore Water	PRIS	saturated	45	Final Cycle	12	8670	1363
Ex1Sbeg1	Soil	PUMP		1	Start		34780	1173

Sample ID	Pore Water or Soil	Site	Dry-Wet Cycle	Sampling Day	Time	Core Number	Number of Reads	Number of OTUs
Ex1Sbeg2	Soil	PUMP		1	Start		32870	1121
Ex1S1	Soil	PUMP	3-day	63	Start	1	44357	1067
Ex1S3	Soil	PUMP	3-day	63	Final Cycle	3	47986	1311
Ex1S4	Soil	PUMP	3-day	63	Final Cycle	4	27117	785
Ex1S5	Soil	PUMP	9-day	63	Final Cycle	5	26000	1072
Ex1S6	Soil	PUMP	9-day	63	Final Cycle	6	23764	1045
Ex1S8	Soil	PUMP	9-day	63	Final Cycle	8	28378	1057
Ex1S9	Soil	PUMP	saturated	63	Final Cycle	9	26287	893
Ex1S10	Soil	PUMP	saturated	63	Final Cycle	10	24569	920
Ex1S11	Soil	PUMP	saturated	63	Final Cycle	11	29440	1188
Ex1S14	Soil	PUMP	unsaturated	63	Final Cycle	14	26177	993
Ex1S15	Soil	PUMP	unsaturated	63	Final Cycle	15	27426	1011
Ex1S16	Soil	PUMP	unsaturated	63	Final Cycle	16	21671	415
Ex2Sbeg1	Soil	PRIS		1	Start		27774	2381
Ex2Sbeg2	Soil	PRIS		1	Start		9036	1380
Ex2Sbeg3	Soil	PRIS		1	Start		29631	2089
Ex2S2	Soil	PRIS	3-day	45	Final Cycle	2	29011	2048
Ex2S3	Soil	PRIS	3-day	45	Final Cycle	3	33106	1848
Ex2S4	Soil	PRIS	3-day	45	Final Cycle	4	15253	1701
Ex2S5	Soil	PRIS	9-day	45	Final Cycle	5	22871	1885
Ex2S6	Soil	PRIS	9-day	45	Final Cycle	6	18099	1862
Ex2S8	Soil	PRIS	9-day	45	Final Cycle	8	25361	1996
Ex2S10	Soil	PRIS	saturated	45	Final Cycle	10	22252	1994
Ex2S11	Soil	PRIS	saturated	45	Final Cycle	11	15630	1527
Ex2S12	Soil	PRIS	saturated	45	Final Cycle	12	47804	2499
Ex2S14	Soil	PRIS	unsaturated	45	Final Cycle	14	23635	1923
Ex2S15	Soil	PRIS	unsaturated	45	Final Cycle	15	20064	1822
Ex2S16	Soil	PRIS	unsaturated	45	Final Cycle	16	31645	1987
**TOTAL**							1527190	
Reads							1661009	
chimera							59006	
Singletons							74813	
Clean Reads							1527190	
